# CottonGen: a genomics, genetics and breeding database for cotton research

**DOI:** 10.1093/nar/gkt1064

**Published:** 2013-11-06

**Authors:** Jing Yu, Sook Jung, Chun-Huai Cheng, Stephen P. Ficklin, Taein Lee, Ping Zheng, Don Jones, Richard G. Percy, Dorrie Main

**Affiliations:** ^1^Department of Horticulture, Washington State University, Pullman, WA 99164-6414, USA, ^2^Cotton Incorporated, Cary, NC 27513, USA and ^3^Crop Germplasm Research Unit, USDA-ARS-SPARC, College Station, TX 77845, USA

## Abstract

CottonGen (http://www.cottongen.org) is a curated and integrated web-based relational database providing access to publicly available genomic, genetic and breeding data for cotton. CottonGen supercedes CottonDB and the Cotton Marker Database, with enhanced tools for easier data sharing, mining, visualization and data retrieval of cotton research data. CottonGen contains annotated whole genome sequences, unigenes from expressed sequence tags (ESTs), markers, trait loci, genetic maps, genes, taxonomy, germplasm, publications and communication resources for the cotton community. Annotated whole genome sequences of *Gossypium raimondii* are available with aligned genetic markers and transcripts. These whole genome data can be accessed through genome pages, search tools and GBrowse, a popular genome browser. Most of the published cotton genetic maps can be viewed and compared using CMap, a comparative map viewer, and are searchable via map search tools. Search tools also exist for markers, quantitative trait loci (QTLs), germplasm, publications and trait evaluation data. CottonGen also provides online analysis tools such as NCBI BLAST and Batch BLAST.

## INTRODUCTION

Cotton (*Gossypium spp*.) is the world’s leading natural textile fibre crop and a significant contributor of oilseed. Consisting of 50 species with different levels of ploidy, *Gossypium* has long served as a model for studying fundamental biological questions on genome evolution, plant development, polyploidization and crop productivity (1–5). The application of new sequencing technologies and high-throughput genotyping has improved understanding of diploid and polyploid cotton species and has resulted in a wealth of genetics, genomics and breeding information for cotton over the last two decades. These publicly available resources include 49 genetic maps, 24 000 markers, >1000 quantitative trait loci (QTL) representing >30 agronomically important traits, phenotype data from >15 000 germplasm accessions, >650 000 NCBI sequences derived from 181 DNA libraries, 18 000 genes and gene products, 460 000 expressed sequence tags (ESTs) and expression data in the form of microarrays and RNA-Seq from high-throughput sequencing. More recently, two genome assemblies and annotations of *Gossypium raimondii*, have become available (6,7). The availability of the cotton genome sequence provides a major source of candidate genes with potential for the genetic improvement of cotton quality and productivity. Integrating this whole genome data with other genomic and genetic data in an online database that is easy to query, view and download is essential to maximize utility of these valuable research data.

Three online databases traditionally hosted much of the available genomic and genetic cotton data prior to 2012. CottonDB (8) was founded in 1995 as part of a national USDA-ARS program to develop plant genome databases for all agricultural commodities. Using a hybrid database system, the genomic, genetic, taxonomic and bibliographic data were stored in an object-oriented AceDB database (9), while the genetic maps and genome sequences were maintained in a MySQL relational database. Initiated in 2004, the Cotton Marker Database (CMD) (10) was funded by Cotton Incorporated to provide centralized access to all publicly available cotton simple sequence repeat (SSR) markers and accelerate basic and applied research in molecular breeding and genetic mapping. It used a custom MySQL database with search interfaces developed in the Perl programming language. The third database, TropGene Cotton (11), was developed as part of a larger project to manage genetic, molecular and phenotypic data on tropical crop species. It uses a custom MySQL database with search interfaces developed in the Java programming language. The majority of public cotton data from TropGene was shared with CottonDB. CottonDB, while rich in data, was limited by older technology, which resulted in a relatively unfriendly query interface and made further development difficult. CMD, although more user friendly, was limited primarily to marker data and used a custom database schema that limited the integration of other types of data. CottonGen, therefore, was created to address these limitations by consolidating and expanding cotton data from CottonDB, CMD and TropGene into a new, standards-based, freely accessible scientific database for worldwide cotton research. Another feature developed in CottonDB but adopted by CottonGen is the hosting of the website for the International Cotton Genome Initiative (ICGI). ICGI is a non-profit organization created in 2000 to increase knowledge of the structure and function of the cotton genome for the benefit of the global community. It facilitates global communication, collaboration, and education; knowledge and resource integration; technology and resource development; and coordinates research planning. The CottonGen team agreed to redevelop and host the ICGI website within CottonGen as part of its mission to serve as a centralized resource for the cotton community.

CottonGen is developed using Tripal (12), a toolkit for construction of online genomic and genetic databases. Tripal is based on a community-derived database schema named Chado (13) and employs the use of controlled vocabularies such as the Sequence Ontology (14), Gene Ontology (15) and others to ensure standardization of data storage. Tripal currently is used for several genome databases (16–21). Additionally, Tripal provides simplified site development by merging the power of Drupal (http://drupal.org), a popular web Content Management System allowing non-programmers the ability to contribute content with Chado.

Migration of data from CottonDB to CottonGen was initiated on 1 October 2011, and CottonGen was released one year later, superseding CottonDB and CMD with additional data and enhanced functionality. As of 15 August 2013, CottonGen includes (i) the *Gossypium raimondii* whole genome assemblies and annotation, (ii) annotated unigene for the *Gossypium* genus, (iii) extensive genetic and QTL maps, markers and traits, (iv) trait evaluation data, (v) enhanced user interfaces including various search tools with downloadable results and (vi) resources to support community activities and to facilitate communication among cotton researchers. Here we describe the data and the functionality in CottonGen.

## DATABASE DESCRIPTION

### CottonGen Data and Web Interface

CottonGen contains various genetics, genomics and trait evaluation data including annotated whole genome sequences, EST sequences, markers, traits, genetic maps, genes, taxonomy, germplasm and publications. All CottonGen web pages have a common navigation menu for easy access. The navigation menu provides links for general information, data, search, tools, help and community resources for the ICGI. The data section lists major data classes in CottonGen (Table 1), such as gene, genome, germplasm, map, marker, publication, species and trait. Users can view a summary of the data, and various links to access the data. The search section lists various search tools such as for genes, germplasm, markers, QTL, publications and trait evaluation. Each search tool provides options for customization by applying restrictions in the query. From the search result site or the downloads page, users can download the entire data and/or go to the various data details pages. Major CottonGen data and the web interface to the data are described below.

**Table 1.** Number of CottonGen entries by data type (15 August 2013)

Data type	Number of entries	Details
BLAST	20	5 peptide data sets, 15 nucleotide data sets (genome sequences, markers, unigenes, ests) for BLAST searching.
Genome	2	Draft BGI v1.0 and JGI annot v2.1 *G. raimondii* genome projects.
Gene	119 971	1269 cotton genes from NCBI gene (06/12/2013); 40 976 and 77 726 CDS from *the* BGI v1.0 and JGI annot v2.1 *G. raimondii* genome projects, respectively, and 21 698 Contigs from CottonGen Gossypium Unigene v1.0.
Germplasm	14 959	From 14 collections.
Marker	23 935	19 074 SSRs, 3541 RFLPs, 2146 AFLPs, 1018 SNPs and 310 other types.
Map	49	34 559 loci
QTL	988	Representing 25 traits
Publication	10 731	Journal articles, conference proceedings, patents, book chapters and theses.
Species	49	Origin, genome group, germplasm, haploid number, sequences and libraries.
Trait evaluation	73 296	From 6871 accessions

### Genomics data

#### Whole genome sequence data

**Figure 1.**
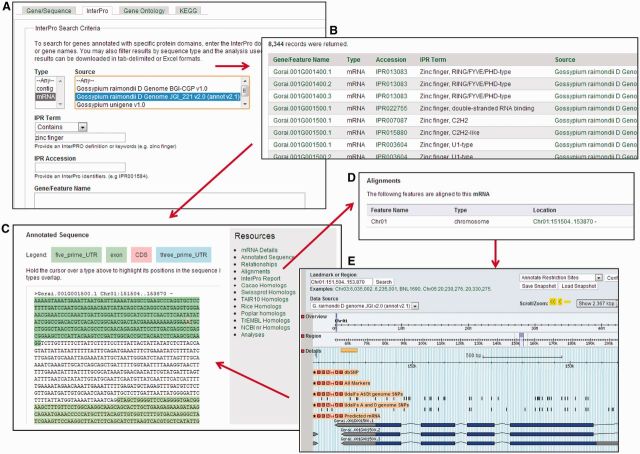
Gene/Sequence search site in CottonGen. (**A**) Genes/sequences can be searched using various categories, such as by name, GO terms, InterPro protein domain name or KEGG pathway term. The example shows the InterPro term search site. (**B**) The search result page has links to the download, gene/sequence detail page and external database. (**C**) The Gene detail page has various tabs to show the data. The annotated sequence page is highlighted. (**D**) The alignment tab of the gene detail page shows the position in the whole genome with link to GBrowse. (**E**) The GBrowse page linked from the alignment tab of the gene detail page. Users can go back to the gene detail page from GBrowse.

CottonGen includes the first fully sequenced cotton species, *Gossypium raimondii*, from two independent research teams (6,7). On CottonGen, these assemblies are titled the ‘*Gossypium raimondii* (D5) genome JGI assembly v2.0 (annot v2.1)’ (6) (referred to hereafter as the JGI version) and the ‘*Gossypium raimondii* (D5) Draft Genome BGI-CGP v1.0 Assembly & Annotation’ (7) (referred to hereafter as the draft BGI version). The predicted genes from these assemblies have been further annotated by the CottonGen team to include homology to proteins in other well annotated or closely related species, and *in silico* annotation of InterPro protein domains, GO terms and Kyoto Encyclopedia of Genes and Genomes database (KEGG) pathway terms, providing information on probable pathways and traits. Additional annotation by the CottonGen team includes the alignment of cotton genetic markers, and cotton transcripts such as CottonGen Unigene version v1, Udall cotton Unigene contigs (22), PlantGDB Cotton Unigene and NCBI Cotton ESTs from all major *Gossypium* species. Single nucleotide polymorphisms (SNPs) between the diploid genomes of A and D and those between the tetraploid genomes of AT and DT (T represents tetraploid) were also aligned to the JGI version of the *G. raimondii* reference genome (23,24). The annotated sequence data can be accessed in CottonGen via the genome page, gene and sequence search tools and GBrowse (25). The genome pages, found under the data navigation menu, contain various downloadable files including the FASTA files of predicted gene transcripts, coding sequences (CDS) and predicted gene peptides. Excel files of protein homologues with cotton genes and other species including those found in databases such as Swiss-Prot and TrEMBL (26) and NCBI nr (27), are also available with hyperlinks to these databases. Other downloadable files include ESTs and genetic markers in FASTA and Excel format that map to the whole genome sequences and functional annotation files from protein, Interpo and KEGG alignments. In the gene and sequence search tools, whole genome data can be found by filtering by name, GO terms, InterPro domains or KEGG pathway terms (28) (Figure 1). From the alignment page, users can go to GBrowse. Using GBrowse, site visitors can view genomic features aligned to the genome, such as gene models, repeats, SNPs, as well as alignments of ESTs, repeats, genetic markers and genes from other plant model species. Each feature in GBrowse is hyperlinked to a page with sequences and additional information, and hyperlinks to external databases where applicable. The chloroplast genome sequences and annotations of *Gossypium hirsutum*, *Gossypium barbadense*, *Gossypium arboreum* and *G. raimondii* are also available in GBrowse.

#### Annotated EST unigene

CottonGen contains all *Gossypium* ESTs publicly available from dbEST at NCBI as of 12 September 2012. To reduce inherent redundancy in ESTs and generate a data set representing the genes of cotton, we developed the CottonGen v1.0 unigene. Routine processing involved sequence filtering for contamination against the NCBI UniVec database and species-specific chloroplast, mitochondrial, tRNA and rRNA sequences using the BLAST algorithm with NCBI UniVec-recommended parameters; trimming of low quality sequence; assembly into contigs using CAP3 (29) with an overlap percentage parameter of 90% (p -90); and annotation. 437 185 filtered sequences were assembled into 21 698 contigs and 128 218 singletons to make a unigene set of 149 916 sequences. The CottonGen annotation procedure includes comparison of both the filtered ESTs and the EST contig consensus sequences using BLASTX against the SWISS-PROT, TrEMBL, InterPro, TAIR (30) and other well annotated species protein databases. The top 10 matches with an expectation value <1e-6 are recorded for each EST and contig. Results of *in silico* functional annotations of Gene Ontology (GO) terms and functional classification by pathways from KEGG are also recorded in the database. The 21 698 contigs from the v1.0 unigene can be searched using the gene and sequence search tools by name, Interpro domain, GO term or KEGG term or gene and the results downloadable as Excel files from the search page. All the unigene data set and annotations can also be obtained from the downloads page. Additional sequence annotation includes computational analysis of SSR found in the unigene contigs using the method described in Jung et al., 2008. Of the 21 698 contigs, 24.6% had one or more SSRs, with 493 motifs detected in 6979 SSRs. The results may be obtained from the Downloads page as an Excel file with details for each SSR containing sequence including motif, motif length, location in the sequence, location relative to the ORF, suggested primers and expected product size.

#### NCBI genes

All *Gossypium* sequences from the NCBI nucleotide database were downloaded, parsed for gene, mRNA, CDS, 5′UTR and 3′UTR features and imported to CottonGen. As with predicted genes from whole genome sequences, genes parsed from NCBI have been further annotated by homology to genes in other species, InterPro protein domains, GO terms and KEGG pathway terms. The distinct gene names in *Gossypium* are stored separately in the database to build a community-driven gene database for cotton. Each gene, unique in the *Gossypium* genus, is currently linked to all the NCBI genes from various species and will serve as a base entity to be linked to other associated data such as predicted genes from whole genome sequences, QTL, genetic markers and mutant phenotypes as annotation progresses. All genes and mRNAs that are parsed out from NCBI sequences are searchable in the gene search site.

### Map, marker and QTL data

**Figure 2.**
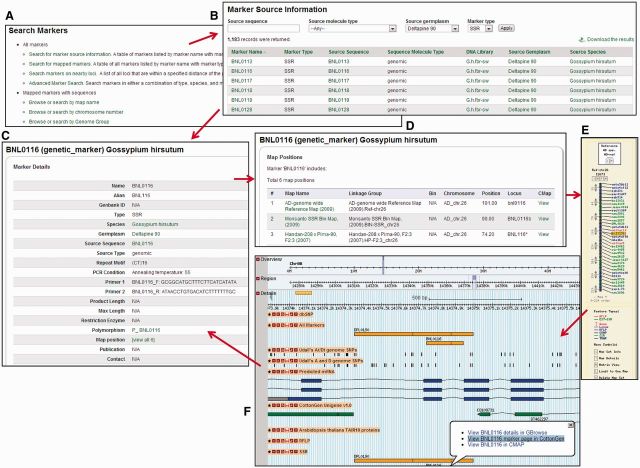
Marker search site in CottonGen. (**A**) Multiple markers search sites are available based on the type of information users are interested in. (**B**) An example search interface where users can view and search for marker source information. (**C**) A Marker details page with various links to detailed information. (**D**) The Map position tab of the marker page shows all the maps where the marker has been mapped. (**E**) From the marker page users can go to the CMap. (**F**) For the markers that are anchored to the genome, CMap provides hyperlinks to GBrowse. From GBrowse users can follow the links to go back to Cmap, the marker detail page or the Sequence Retrieval Tool.

CottonGen provides access to the cotton genetic, QTL, and physical (FPC) maps, including the underlying molecular markers, QTL and mapping populations. For sequence-based markers such as SSRs, Amplifed Fragment Length Polymorphisms (AFLPs), Sequence Related Amplified Polymorphisms (SRAPs), and cDNA-Rapid Fragment Length Polymorphisms (RFLPs), CottonGen provides details on experimental conditions, such as the primer, amplicon-sequence information and the PCR amplification conditions. CottonGen currently has 49 maps, which covers *Gossypium* genome groups AD, A, D and G, consisting of approximately 34 000 marker loci and a thousand QTLs. Markers can be browsed and searched using various search interfaces (found under search and then markers in the navigation menu). All markers can be searched by marker source, map information or nearby loci. The advanced marker search interface allows researchers to search by various categories in combination (Figure 2). Researchers can also browse/search only the mapped markers with sequences using various categories. From the search result page, researchers can go to the details pages of markers, maps, sequences, germplasm and species. From the marker details page, relevant data such as marker source, primers, polymorphisms, map information and anchored position in the genome can be accessed.

CottonGen houses 273 QTLs with associated data such as CottonGen curator-assigned QTL label, published symbol, trait name, alias, population, map position, associated markers and statistical values. The QTL search page allows searching for QTLs by trait name, published symbol and QTL label. Search results are hyperlinked to CMap (31) and downloadable in Excel format.

### Germplasm and trait evaluation data

**Figure 3.**
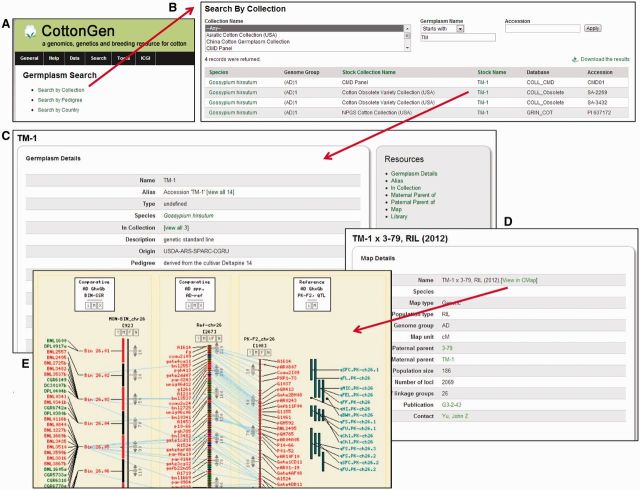
Germplasm search site in CottonGen. (**A**) Multiple germplasm search sites are available based on the type of information users are interested in. (**B**) An example search where users can view and search for germplasm and their collection centre. (**C**) A germplasm details page with various tabs to show the detailed information. (**D**) The Map tab of a germplasm page shows all the maps for which the germplasm has been used. (**E**) From the map page users can open CMap for further exploration.

CottonGen includes information for each of the 50 *Gossypium* species such as genome groups, geographic origins, inter-species compatibilities and germplasm. About 15 000 germplasm accessions are stored in CottonGen. These individuals were identified from >47 000 entries that consist mainly of the USDA-ARS Germplasm Resources Information Network (32) cotton collection, the cotton germplasm collection of the China Cotton Research Institute, the Chinese Academy of Agricultural Sciences and the cotton germplasm collection of Uzbekistan Center of Genomics and Bioinformatics, Academy of Sciences of Uzbekistan. Germplasm data include aliases, pedigrees, publically available passport information, stock collection centre, associated maps, libraries and sequences. In addition, trait evaluation data, with >118 000 trait scores, from ∼9000 germplasm are available. The *Gossypium* species summary page (found under data and then species in the navigation menu) provides a list of species along with information such as genome group, haploid chromosome number and geographic origin. The summary of data available in CottonGen is also given: number of germplasm, sequences and DNA libraries. The species name in the table leads to a species page, which shows more details such as common name, images and additional data as seen in the summary table. The species page also shows the results of functional analysis of the genes, both from NCBI and whole genome sequences, which include KEGG and GO analysis reports. Several germplasm search pages provide access to different types of data (Figure 3). The search by collection page provides a list of germplasm along with stock collection centre information. The search can be filtered by collection centre name, germplasm name and/or accession name in the stock centre. The search by pedigree page provides an interface to search germplasm by pedigree and the search germplasm by country page searches by the country of origin. From the germplasm search page, researchers can go to the germplasm details page, which shows all the detail information such as pedigree, passport, collection centre, image and associated genotypic and phenotypic data. Germplasm can also be searched based on their trait evaluation data. Both the qualitative and quantitative trait evaluation search sites allows the trait values of up to three trait descriptors to be specified to view the germplasm trait data. Data from all the search result sites can be downloaded in Excel files.

### Publications

CottonGen houses information about publications that are important to cotton researchers. Details about publications were imported to CottonGen from NCBI PubMed (http://www.ncbi.nlm.nih.gov/pubmed) and the USDA National Agricultural Library (NAL) (http://agricola.nal.usda.gov/) databases. Additionally, details of publications from other journals not present in PubMed or the USDA NAL databases were manually imported to CottonGen. In addition, CottonGen maintains reference information and abstracts for works published in cotton research conference proceedings such as the ICGI Conferences and the Plant and Animal Genome Conferences. Book chapters, theses and patents are also collected. In total, CottonGen houses 10 731 references. Publications can be found using a combinations of keywords (in the abstract or title), all or partial titles, authors and other categories. Search results link to publication pages that contain the abstract, citation, external link to the full article and other details about the publication.

### Online analysis tools

CottonGen contains several online analysis tools. These include an instance of NCBI’s wwwBLAST tool (http://www.ncbi.nlm.nih.gov/staff/tao/URLAPI/wwwblast/) and a custom Batch BLAST tool where users can perform pair-wise BLAST alignments using their sequences against the current 20 CottonGen data sets. The Batch BLAST server supports upload of large data sets for pair-wise comparison. It executes BLAST, and parses the output into an Excel file. Users are notified by email when the job is complete and directed to a website to download result files. The same data sets are available in both BLAST servers for alignment. Protein data sets available for BLAST include *Gossypium* proteins from GenBank and UniProKB and *G. raimondii* protein sequences from the draft BGI v1.0 and JGI v2.1 genome data. Nucleotides sequence databases include GenBank *Gossypium* sequences, *Gossypium* dbSNP, CottonGen SSR, RFLP, and SNP/InDel marker sequences, CottonGen *Gossypium* unigene v1.0, DFCI Cotton Gene Index v11 (http://compbio.dfci.harvard.edu/tgi/plant.html), PlantGDB (http://www.plantgdb.org/) unigene from several *Gossypium* species, Udall 2012 transcript contigs and predicted genes and genome sequences from the BGJ and JGI genome data. The Sequence Retrieval tool enables download of sequences including full chromosomes, scaffolds, genes, full transcripts, transcript coding sequences, proteins, genetic markers aligned to chromosomes, unigene contigs and ESTs. Users supply a list of sequence names to retrieve, and can filter by a specific genome assembly, unigene or other project data. For features aligned to a whole genome, such as genes, transcripts and genetic markers, a user can include a specified number of upstream and downstream bases in the sequence.

### Community resources

CottonGen houses the resources for the ICGI. It maintains the ICGI membership database, information for the ICGI biennial international research conferences, hosting of biennial elections and tools for registration and manuscript submission for the 2012 ICGI Conference. The CottonGen home page includes rotating pictures for recent research stories or community news, brief project descriptions, a news section for the cotton community and a section to quickly find newly added site functionality or data. Email mailing lists for both CottonGen and ICGI are available for communication with the community, and the mailing list archives can be viewed online. Other resources in the help section provide a Frequently Asked Question page for CottonGen and ICGI and user tutorials for both.

## FUTURE PLANS

CottonGen will be updated as new data become available and new or improved functionality is added to the site. This includes adding GBrowse-syn, a GBrowse-based synteny browser (33), to view multiple sequence alignment data, synteny or co-linearity data from closely related or useful species such as cacao and Arabidopsis. A comprehensive breeders toolbox, similar to that developed for the Rosaceae community as part of the USDA NIFA SCRI-funded project RosBREED (Grant number #2009-51181-06036), is planned for future implementation. In addition, a digital image library will be created for over one hundred thousand images created from the USDA-ARS Research Project: ‘Genotypic and Phenotypic Analysis and Digital Imaging of Accessions in the US National Cotton Germplasm Collection’. The associated phenotypic data will also be stored in CottonGen.

## CONCLUSION

CottonGen is now the consolidated cotton genomics, genetics and breeding database for the cotton community. It aims to provide a comprehensive, integrated, online resource that serves basic, translational and applied cotton research. It is constructed using the open-source Tripal genome database toolkit, which merges the power of Drupal, a popular web Content Management System with that of Chado, a community-derived database schema for storage of genomic and genetic data. Data types in CottonGen include maps and markers, whole genome assemblies and annotations, gene and sequences with analyzed data, taxonomic and germplasm data and publication data. CottonGen maintains online resources for ICGI, a non-profit organization created as a global affinity group with common goals and interests. From its release on 1 March 2012 to 15 August 2013, CottonGen had 11 111 visits by 4756 unique visitors from 94 countries who accessed 75 551 pages.

## FUNDING

Cotton Incorporated; the USDA-ARS Crop Germplasm Research Unit at College Station, TX; Southern Association of Agricultural Experiment Station Directors; Bayer CropScience; Dow/Phytogen; Monsanto. Components of the infrastructure for CottonGen were created under funding for Tripal development for other databases (USDA NIFA [2009-51181-06036, 2009-51181-05808]). As these databases all use the same underlying Tripal infrastructure, source code was shared amongst all of these databases. That code is also freely available on the Tripal website at http://tripal.info. Funding for open access charge: CottonGen Grant.

*Conflict of interest statement*. None declared.
